# Multimorbidity of Psoriasis: A Large-Scale Population Study of Its Associated Comorbidities

**DOI:** 10.3390/jcm13020492

**Published:** 2024-01-16

**Authors:** Manuel Almenara-Blasco, Tamara Gracia-Cazaña, Beatriz Poblador-Plou, Clara Laguna-Berna, Jonás Carmona-Pírez, Alba Navarro-Bielsa, Alexandra Prados-Torres, Antonio Gimeno-Miguel, Yolanda Gilaberte

**Affiliations:** 1 Department of Dermatology, Miguel Servet University Hospital, IIS Aragon, Paseo Isabel la Católica 1-3, 50009 Zaragoza, Spain; manuelalmenarablasco@gmail.com (M.A.-B.);; 2 EpiChron Research Group, Aragon Health Sciences Institute (IACS), Aragon Health Research Institute (IIS Aragón), Miguel Servet University Hospital, 50009 Zaragoza, Spain; 3 Research Network on Chronicity, Primary Care, and Health Promotion (RICAPPS), Institute of Health Carlos III (ISCIII), 28029 Madrid, Spain; 4 Subdirección Técnica Asesora de Gestión de la Información, Andalusian Health Service, 41071 Sevilla, Spain

**Keywords:** psoriasis, comorbidities, epidemiology

## Abstract

Introduction: Psoriasis is a chronic disease of the skin with a prevalence of 2% in the general population. The high prevalence of psoriasis has prompted the study of its comorbidities in recent decades. We designed a study to determine the prevalence of psoriasis in a large-scale, population-based cohort, to exhaustively describe its comorbidities, and to analyze which diseases are associated with psoriasis. Methods: Retrospective, observational study based on the clinical information contained in the electronic health records of the individuals in the EpiChron Cohort with a diagnosis of psoriasis (31,178 individuals) in 2019. We used logistic regression models and calculated the likelihood of the occurrence of each comorbidity based on the presence of psoriasis (*p*-value < 0.05). Results: The prevalence of psoriasis was 2.84%, and it was more prevalent in men (3.31% vs. 2.43%). The most frequent chronic comorbidities were disorders of lipid metabolism (35.87%), hypertension (35.50%), and other nutritional-endocrine-metabolic disorders (21.79%). The conditions most associated with psoriasis were (odds ratio; 95% confidence interval) tuberculosis (2.36; 1.24–4.49), cystic fibrosis (2.15; 1.25–3.69), amongst others. We did not find a significant association between psoriasis and hypertension or neoplasms (0.90; 0.86–0.95). Conclusions: This study revealed significant associations between psoriasis and cardiac, psychological, and musculoskeletal comorbidities.

## 1. Introduction

Psoriasis is a chronic inflammatory disease of the skin; its pathogenesis is diverse and is influenced by genetic and environmental factors [1,2]. It is the most frequent immune-mediated skin disease, with a prevalence rate of around 2% in the general population [3,4]. There are important differences in its prevalence depending on the origin of the population, being higher in countries such as the United States and Canada, where it is around 5%, and lower in Asia, where the prevalence ranges between 0.4 and 0.7% [4,5]. In terms of severity, it is estimated that 27% of patients with this condition have severe psoriasis, while the rest present mild or moderate forms [6]. Psoriasis can begin at any stage of life; however, there are two peaks of maximum incidence, the first in youth around 20–30 years old and another one in a mature stage around 50–60 years old [4]. It has been described that women have an earlier onset of psoriasis than men [4,5].

Clinically, the disease occurs in flares and is characterized by well-demarcated erythematous plaques with whitish scales of variable distribution [6]. There may be various clinical forms, such as pustular psoriasis, guttate psoriasis, and erythrodermic psoriasis, among others. Histologically, psoriasis is characterized by hyperkeratosis with parakeratosis, acanthosis of the epidermis, dilated vessels, and a perivascular inflammatory infiltrate with a predominance of lymphocytes [6].

While the skin lesions caused by psoriasis are the most visible symptoms of the disease, psoriasis is often associated with several other health conditions, known as psoriasis comorbidities [7,8]. Research has connected psoriasis to various other illnesses, including psoriatic arthritis, type 2 diabetes, metabolic syndrome, lung disease, non-alcoholic fatty liver disease (NAFLD), uveitis, and mental health conditions. However, the relationship between psoriasis and some illnesses, such as neoplasms and multiple sclerosis, is still unclear [9]. While diet and obesity are thought to play a role in some psoriatic complications, it is also demonstrated that psoriasis is a systemic inflammatory condition that can cause atherosclerosis and increase the risk of cardiovascular (CVD) and cerebral diseases [10,11].

It is essential to enhance our understanding of comorbidities accompanying psoriasis to provide optimal patients’ care, which should take into account the presence of multiple comorbidities. The comprehensive analysis of the comorbidity profile of individuals with psoriasis, including not only the most common conditions but also those that are consistently linked to psoriasis regardless of their frequency, might shed light on the connections between this condition and other diseases. This information could help to develop personalized and comprehensive care plans for patients with psoriasis that consider all of their health concerns.

The objective of this study is to determine the prevalence of psoriasis in a large-scale, population-based cohort in the Spanish region of Aragon, to exhaustively describe its comorbidities, and to analyze which diseases are systematically associated with the presence of psoriasis.

## 2. Methods

### 2.1. Study Design and Population

We conducted a retrospective observational study in the EpiChron Cohort, which links socio-demographic and clinical data from all the users of the public health system in the Spanish region of Aragón. This cohort is based on the information registered in the electronic health records (EHRs) and clinical–administrative databases of approximately 98% of the inhabitants of Aragón (reference population: 1.3 million people). For this analysis, we included all the 31,178 patients from the cohort with a diagnosis of psoriasis in 2019. Our research group has conducted other studies on chronic dermatological conditions, such as atopic dermatitis, using a similar methodology [12].

The Clinical Research Ethics Committee of Aragón (CEICA) approved this study (The approval code is ESPI17/0024) on 15 February 2017, and waived the requirement to obtain informed consent from patients given the epidemiological nature of the project, which used anonymized data.

### 2.2. Study Variables

For each participant, we studied sex, age, residence area (rural vs. urban), deprivation index of the area, country of birth, and all chronic conditions and some acute diseases of interest registered in their EHRs. Diagnoses were initially coded using the International Classification of Primary Care, First Edition (ICPC-1) and mapped to the International Classification of Diseases, 9th Edition, Clinical Modification (ICD-9-CM) codes using a codifier system. Then, each ICD-9-CM code was sorted into 226 mutually exclusive clinical categories using the Clinical Classifications Software (CCS) [13]; 153 of them were classified as chronic using the Chronic Condition Indicator (CCI) [14] open-source tool. This tool defines chronic as those conditions with a duration of ≥12 months and that meet at least one of the two following criteria: (a) demand continuous care with a high risk of recurrence, and/or with implications for the management of the patients; (b) have limitations on self-care, independent living, and social interactions. We also included the following ten acute conditions considered clinically relevant by the three clinical experts of the research team (M.A.-B., T.G.-C., Y.G.) after a bibliographic review: unspecified local infection of skin and subcutaneous tissue (staphylococcal, streptococcal; ICD-9-CM 686.9), streptococcal infections of the upper respiratory tract (ICD-9-CM 034.0), other upper respiratory diseases (CCS 134, acute and chronic codes), other upper respiratory infections (CCS 126, except ICD-9-CM 034.0, which was a separate category), allergic rhinitis (ICD-9-CM 477), conjunctivitis (ICD-9-CM 370 and 372.0), acne (ICD-9-CM 706.0 and 706.1), otitis media (ICD-9-CM 381 and 382), external otitis (ICD-9-CM 380.1 and 380.2), and ear wax (ICD-9-CM 380.4). Some of the diagnostic labels were renamed or grouped/ungrouped by clinical experts with minor changes to facilitate their clinical interpretation.

### 2.3. Statistical Analysis

We performed a descriptive analysis of the frequency and prevalence (%) of psoriasis in the study population based on sex, age (i.e., 0–11, 12–17, 18–44, 45–64, and ≥65 years), country of birth, residence area, and deprivation index of the area. We used logistic regression models to calculate the likelihood of presenting psoriasis according to each of the categories of the aforementioned variables in the form of crude and age- and sex-adjusted odds ratios (ORs). Adjusted ORs were compared, setting statistical significance at *p* < 0.05. We then analyzed the socio-demographic characteristics and comorbidity profile of patients affected by psoriasis. Results were calculated as means and/or frequencies with their corresponding standard deviations and/or 95% confidence intervals (CI).

In the analysis of psoriasis comorbidity, we first described the frequency and prevalence of chronic and acute diseases of interest. Then, for the identification of the comorbidities systematically associated with psoriasis, we used logistic regression models and calculated the likelihood of occurrence of each comorbidity (dependent variable) based on the presence of psoriasis (independent variable) as crude and sex- and age-adjusted ORs.

All the analyses were performed in RStudio software (version 1.4.1106, Rstudio, Boston, MA, USA).

## 3. Results

### 3.1. Prevalence and Socio-Demographics of Psoriasis

A total of 31,178 cohort patients had a diagnosis of psoriasis during the study period (mean age 46.54 years, SD 18.40, 45.90% women), resulting in an overall prevalence of 2.84% (Table 1). This prevalence was significantly lower in women compared to men (2.43% vs. 3.31%; OR 0.73, 95% CI 0.71–0.74; Table 1).

Spanish individuals were more frequently affected by psoriasis than those of different nationalities (ORs from 0.21 to 0.82); only North Americans and Western Europeans had a similar prevalence to Spanish people (OR 0.98, 95% CI 0.87–1.10; Table 2). The difference in the prevalence of psoriasis between men and women stood out in the Asian and North African populations, with women having half the prevalence of men: the Asian population (2.82% in men vs. 1.48% in women) and the North African population (2.36% in men vs. 1.35% in women). The prevalence of psoriasis in the population of Sub-Saharan Africa was also striking (0.63%; OR 0.21, 95% CI 0.17–0.27).

Regarding the area of residence, psoriasis was slightly less prevalent in rural areas (2.78% vs. 2.88%; OR 0.96, 95% CI 0.94–0.98). No relevant differences in the prevalence of psoriasis according to the deprivation index of the area were observed.

### 3.2. Comorbidity of Psoriasis

Approximately three in four patients with psoriasis showed multimorbidity, with a mean disease burden of 3.75 (SD 2.97) chronic diseases (Table 3). The most frequent chronic comorbidities in patients with psoriasis of all ages and for both sexes were *disorders of lipid metabolism* (35.87%), *hypertension* (35.50%), and *other nutritional-endocrine-metabolic disorders* (21.79%), among others. All these comorbidities were more prevalent in the population with psoriasis, except for *hypertension* (Table 4). *Acute upper respiratory tract infection* (49.13%), *other upper respiratory diseases (Pharyngitis and tonsillitis)* (14.35%), *wax in the ear* (8.71%), and *otitis media* (5.50%) were among the most prevalent acute conditions in this cohort of patients with psoriasis.

Regardless of their prevalence and after adjusting for sex and age, the conditions most associated with psoriasis were (OR; 95% CI): *tuberculosis* (2.36; 1.24–4.49), *cystic fibrosis* (2.15; 1.25–3.69), and *otitis externa* (1.65; 1.44–1.88; Table 4).

The most relevant endocrine and metabolic comorbidities associated with psoriasis were: *other liver diseases* (1.57; 1.46–1.68), *menopausal disorders* (1.28; 1.22–1.3), *obesity* (1.23; 1.19–1.28), *disorders of lipid metabolism* (1.08; 1.06–1.11), and *diabetes mellitus* (1.05; 1.02–1.09). Among digestive comorbidities, the following were highlighted: *regional enteritis and ulcerative colitis* (1.50; 1.32–1.71) and *diverticulosis-diverticulitis* (1.19; 1.10–1.28). *Alcohol-related disorders* (1.26; 1.16–1.37), *sexual disorders* (1.19; 1.11–1.28), *anxiety disorders* (1.12; 1.09–1.16), and *depression and mood disorders* (1.10; 1.06–1.13) were the neuropsychological conditions most associated with psoriasis. Musculoskeletal comorbidities most frequent in psoriatic patients included *rheumatoid arthritis and related disease* (1.42; 1.28–1.57), *other bone disease and musculoskeletal deformities* (1.39; 1.22–1.57) and *spondylosis* (1.21; 1.17–1.26). Infectious diseases such as *unspecified local infection of the skin and subcutaneous tissue* (1.39; 1.16–1.66), *streptococcal upper respiratory tract infections* (1.26; 1.19–1.34), and *conjunctivitis-keratitis* (1.18; 1.07–1.30) were also significantly more prevalent in the psoriatic population.

On the other hand, there were also some relevant comorbidities that did not show or showed an inverse association with psoriasis, including *peripheral and visceral atherosclerosis* (1.10; 0.99–1.22), hypertension (0.98; 0.96–1.01), and *neoplasms* (0.90; 0.86–0.95), among others.

## 4. Discussion

This study utilized real-world data from 1,098,383 patients in the Spanish public health system to examine psoriasis prevalence. The comorbidities of 31,178 patients diagnosed with psoriasis were exhaustively analyzed, with an emphasis on the most common chronic diseases and those with the strongest association with psoriasis, regardless of their overall frequency.

The prevalence of psoriasis obtained in our population (2.84%) is similar to that found in other studies carried out in Spain (2.90%) [1,4]. Differences in the frequency of psoriasis between men and women are inconsistent across various studies and geographical locations [4]. Some studies found no difference in the frequency of psoriasis between genders [15,16,17,18], while others reported a slightly higher prevalence in females [19]. Some studies also reported a higher prevalence in men compared to women [15,20,21]. In our cohort, it seems that being a woman is less associated with psoriasis. Women only presented a higher percentage of psoriasis (0.72% vs. 0.50%) at younger ages (0–11 years old).

The differences in prevalence between rural and urban populations were minimal, although statistically significant. Previous studies have already demonstrated a higher prevalence of psoriasis in urban areas without pointing to a specific cause [22]. As for socio-economic factors, other studies also found no differences in prevalence [23]. In our case, we used the deprivation index of the area as an aggregated proxy variable for socio-economic status, and the differences found were minimal and difficult to explain since there was no correlation of prevalence with greater or lesser economic status.

To comprehensively understand the comorbidity profile of psoriasis patients, we employed a two-step strategy. Firstly, we outlined the most common comorbidities, regardless of their relationship to psoriasis. Secondly, we highlighted those comorbidities that have an increased probability of occurring in individuals diagnosed with psoriasis. The interplay between psoriasis and these comorbidities is complex and may involve causal, resulting, or shared etiological factors. Our study supports previous research, revealing that patients with psoriasis are frequently plagued by multimorbidity, with almost three in four presenting with multiple diseases and symptoms of different origins.

Previous studies have shown a correlation between psoriasis and altered lipid levels, including decreased HDL levels and increased LDL, VLDL, and triglycerides [24,25]. The prevalence rates of *disorders of lipid metabolism*, the most frequent psoriatic comorbidity in our population, were similar (35.87%) to those reported in other studies (18.9–44%) [24,26,27]. However, the wide range of dyslipidemia percentages indicates that psoriasis is not equally associated with dyslipidemia in all populations, being lower in countries such as Taiwan and higher in the US [24,28]. In addition, dyslipidemia can be worsened by obesity. The association between obesity and psoriasis has been demonstrated in several studies [7,29]. The prevalence of *obesity* in our cohort was 12.21%, very similar to that of other population-based studies in Spain (14.5%) [27]. Other international studies show higher obesity frequencies of up to 20.3% [24].

One of the most common comorbidities associated with psoriasis is type 2 diabetes. Studies have shown that the severity of psoriasis is directly linked to the presence of type 2 diabetes. This is thought to be due to the production of cytokines, such as interleukin-17, which are involved in both psoriasis and type 2 diabetes. The association between diabetes mellitus and psoriasis oscillates in the different studies with ORs between 1.4 and 1.9 [30,31], in our case it is somewhat lower (1.05; 1.02–1.09). Another comorbidity that is often associated with psoriasis is NAFLD [7,32], a condition in which fat accumulates in the liver, causing inflammation and scarring. In our case, we did not directly study NAFLD, but we did observe a significant OR for *other liver diseases* (1.57; 1.46–1.68) containing NAFLD.

Additionally, psoriasis is also associated with an increased risk of CVD, such as heart attacks and strokes. This is thought to be due to the underlying inflammation that is present in psoriasis, which can also contribute to the development of CVD. Hypertension is a risk factor for cardiovascular diseases, and there is a positive association between psoriasis and hypertension [7,9]. However, in our cohort, no statistically significant association between *hypertension* and psoriasis was demonstrated. Nor was a significant association observed with CVD like *peripheral and visceral atherosclerosis*, *other circulatory diseases*, *aortic and peripheral arterial embolism, thrombosis,* or *acute myocardial infarction*. Different studies in populations similar to ours have demonstrated the association between psoriasis and comorbidities integrated into these groups [7,10,28,30]. The findings should raise the question of whether this association is true, whether our population has differential characteristics, or whether the statistical adjustments are different.

Musculoskeletal comorbidities, such as *osteoarthritis, rheumatoid arthritis and related diseases, other bone diseases, musculoskeletal deformities,* and *spondylosis,* were more frequent in psoriatic patients. Among the musculoskeletal pathologies, the comorbidity with the highest association with psoriasis is psoriatic arthritis, with a prevalence of about 25% [33,34]. In our cohort, the diagnosis of psoriatic arthritis would be included in *osteoarthritis,* with a much lower prevalence of around 13.47%, which would indicate that our patients are underdiagnosed.

Psoriasis has been linked to several psychiatric comorbidities, including depression, anxiety, and alcoholism [9,11,35]. The prevalence of these diseases and their OR in patients with psoriasis is always positive when reviewing the literature, but highly variable depending on the type of study and the screening tool used [35,36,37,38]. Our results are somewhat lower than those reviewed for these comorbidities: *alcohol-related disorders*, *sexual disorders*, *anxiety disorders*, *depression, and mood disorders*. This could be due to the fact that the result of our variable is not extracted from a proactive screening but has to have been detected by the patients’ physician.

The relationship between psoriasis and neoplasms has been the subject of much research in recent years. Some studies have found an increased risk of certain types of neoplasms in people with psoriasis, particularly skin, lymphoid, and gastrointestinal tumors [39]. It is believed that the chronic inflammation associated with psoriasis may contribute to the development of certain types of cancer, but more research is needed to confirm this. In our study, the association between psoriasis and *neoplasms* as a whole has not been demonstrated; the prevalence was 5.12% (0.90; 0.86–0.95), very similar to other research (4.78%) [39].

Several studies show an increased risk of cutaneous colonization by *S. aureus* and severe skin infections in patients with psoriasis treated with systemic drugs [40,41]. However, they do not report the prevalence of acute skin infections on a population basis. In our case, they were statistically significant: *otitis externa*, an *unspecified local infection of the skin and subcutaneous tissue*, *streptococcal upper respiratory tract infections,* and *conjunctivitis-keratitis*. These findings should lead us to pay more attention to the examination of the patient with psoriasis.

Finally, if we analyze the comorbidities most associated with psoriasis, they are: *tuberculosis* (2.36; 1.24–4.49), *cystic fibrosis* (2.15; 1.25–3.69), and *otitis externa* (1.65; 1.44–1.88). Before discussing the association between psoriasis and *tuberculosis*, some clarifications regarding the study methodology need to be addressed. The study was conducted using population-based data, and therefore, we cannot ascertain the individual treatment received by patients who developed *tuberculosis*. Moreover, the diagnosis of *tuberculosis* might result from positive serological screening tests and Mantoux tests carried out on psoriasis patients about to undergo immunosuppressive therapy. This diagnosis of latent *tuberculosis* could falsely increase the association with psoriasis and *tuberculosis* compared to the healthy population. A recent study on the Spanish cohort of BIOBADADERM reflects that in the population of patients with moderate to severe psoriasis in Spain, 20.5% of patients exposed to biological treatments were diagnosed with latent *tuberculosis* before starting the biological treatment [42]. The prevalence of latent *tuberculosis* in our sample (0.03%) is much lower because it also includes patients with mild psoriasis, who comprise the majority.

The direct association between *cystic fibrosis* and psoriasis has not been explicitly described. Studies have been conducted on gene promoters to investigate if *cystic fibrosis* is associated with genetically inflammatory diseases, and in the case of psoriasis, no association was found [43]. However, some similarities in immunopathogenic pathways or aggravating factors have been reported in both conditions. For example, *S. aureus* is a microorganism described as a colonizer in both diseases and an aggravating or triggering factor for them [40,44,45]. The presence of *S. aureus* may contribute to skin inflammation along the Th17 axis in patients with psoriasis, and IL-17 is the major effector cytokine in the pathogenesis of this disease [40,46]. The case of a patient presenting both psoriatic arthropathy and *cystic fibrosis* with poor response to treatments has been reported. There was suspicion that the cause might be due to the hyperactivation of Th17 lymphocytes triggered by the colonization of the respiratory tract by microorganisms [47].

Defensins and their associated antimicrobial peptides are fundamental components of the innate immune system [48]. Inherited fluctuations in defensin gene expression could potentially contribute to susceptibility to several conditions, including psoriasis, *otitis media,* and *cystic fibrosis* [49]. This alteration in defensins could help to explain the association of psoriasis with *otitis media* and *cystic fibrosis* in our study.

In patients with psoriasis, elevated levels of hBD2 and LL-37 have been observed in the skin, which may contribute to chronic inflammation and propagate inflammation through the TLR9-IFN connection [49]. Furthermore, the increased expression of hBD2 and LL-37 in the skin of psoriasis patients has been found to be a result of chronic inflammation, and the expression of hBD2 and inflammation may form a positive feedback loop in patients with psoriasis [49]. In the context of *cystic fibrosis*, defensins have been observed to play a pivotal role in the disease’s pathophysiology. Within the realm of *cystic fibrosis*, defensins, particularly hBD1, are consistently expressed throughout the respiratory tract, indicating a significant role in defending against bacterial colonization [49]. Nevertheless, it has been noted that hBD1 expression might be associated with colonization by *Pseudomonas aeruginosa*, a common pathogenic bacterium in *cystic fibrosis* patients [49]. Finally, regarding *otitis externa*, we found cases of external otitis with psoriatic features have been described in 18% of patients with psoriasis, making its diagnosis complex [50]. Additionally, the frequent involvement of the ears in patients with psoriasis could imply an alteration of the epidermal barrier, which could favor superinfection by *S. aureus* [51]. We did find an association between middle ear infections and psoriasis based on mechanisms similar to those in *cystic fibrosis*. It has been demonstrated that defensins hBD1 and hBD2 exhibit antimicrobial activity against *otitis media* pathogens, and the dysfunction of these defensins in patients with psoriasis could potentially increase their susceptibility to suffering *otitis media* [52,53].

Our findings have the potential to improve the comprehension of psoriasis comorbidities and enhance its management for better patient outcomes. However, it is important to note that correlation does not imply causation, and these results should be approached with caution. While some of the connections we discovered align with previous research, others were not previously documented and may warrant further investigation to uncover the underlying biological links between psoriasis and various conditions.

The main strength of our work lies in its large-scale, population-based nature, as it included almost every patient with a psoriatic diagnosis belonging to the reference population area. Moreover, psoriasis comorbidities were exhaustively analyzed through the study of virtually all chronic diseases diagnosed in both primary and hospital care, not just the most prevalent or relevant ones. The use of EHRs guaranteed the reliability of the data, which underwent continuous quality control revisions to ensure its accuracy. The main limitation of our study was its cross-sectional nature, which made determining the chronological order of appearance of diseases impossible, thus hindering the identification of cause and-effect relationships between psoriasis and its comorbidities.

## 5. Conclusions

The present study highlights the high prevalence of psoriasis in the general population and its strong association with various non-dermatologic conditions, including musculoskeletal, neuropsychiatric, cardiometabolic, and infectious conditions, among others. These findings emphasize the need to monitor the comorbidities associated with psoriasis in order to improve its clinical management and prevent its development.

Future longitudinal studies are encouraged to further explore the underlying mechanisms involved in these associations and to provide further insight into the complex nature of psoriasis. Overall, the study highlights the importance of considering the full range of comorbidities in the clinical management of psoriasis to achieve better patient outcomes.

## Figures and Tables

**Table 1:** Frequency and prevalence (%) of psoriasis in 2019 in the EpiChron Cohort (Aragon, Spain) according to sex, age, nationality, area of residence, and deprivation index.

	Men (*n* = 509,506)	Women (*n* = 588,877)	Total (*n* = 1,098,383)
Psoriasis Prevalence	*n* (%)	*n* (%)	*n* (%)
Sex	16,866 (3.31)	14,312 (2.43)	31,178 (2.84)
Age (years)			
0–11	310 (0.50)	395 (0.72)	705 (0.61)
12–17	496 (2.18)	650 (2.24)	1146 (2.22)
18–44	6747 (3.94)	5755 (2.72)	12,502 (3.26)
45–64	6281 (3.79)	4965 (2.78)	11,246 (3.27)
≥65	3032 (3.45)	2547 (2.21)	5579 (2.75)
Nationality			
Spain	15,450 (3.44)	13,079 (2.57)	28,529 (2.98)
Eastern Europe	519 (2.95)	531 (2.13)	1050 (2.47)
Asia	81 (2.82)	42 (1.48)	123 (2.15)
North Africa	240 (2.36)	117 (1.35)	357 (1.89)
Sub-Saharan Africa	53 (0.79)	15 (0.37)	68 (0.63)
Latin America	362 (1.95)	397 (1.18)	759 (1.45)
EU and North America	161 (3.31)	131 (2.54)	292 (2.92)
Area of residence			
Urban	10,013 (3.36)	8961 (2.48)	18,974 (2.88)
Rural	6853 (3.24)	5351 (2.35)	12,204 (2.78)
Deprivation index ^1^			
Q_1_	4527 (3.41)	3945 (2.49)	8472 (2.91)
Q_2_	4072 (3.28)	3504 (2.43)	7576 (2.82)
Q_3_	3643 (3.36)	3067 (2.51)	6710 (2.91)
Q_4_	4624 (3.21)	3794 (2.31)	8418 (2.73)

^1^ Deprivation index of the residence area according to 26 socio-economic indicators and categorized from least (Q_1_) to most (Q_4_) deprived.

**Table 2:** Likelihood of presenting psoriasis based on sex, age, nationality, area of residence, and deprivation index of the area, calculated using logistic regression models.

Variable	Crude OR ^1^	*p*-Value	Adjusted OR ^2^	*p*-Value
Sex				
Men	ref.			
Women	0.73 (0.71–0.74)	0.000		
Age (years)				
0–11	0.18 (0.17–0.19)	0.000		
12–17	0.67 (0.63–0.71)	0.000		
18–44	ref.			
45–64	1.00 (0.97–1.03)	0.968		
≥65	0.84 (0.81–0.86)	0.000		
Nationality				
Spain	ref.		ref.	
Sub-Saharan Africa	0.21 (0.16–0.26)	0.000	0.21 (0.17–0.27)	0.000
Asia	0.72 (0.60–0.86)	0.000	0.76 (0.64–0.91)	0.003
Eastern Europe	0.82 (0.77–0.88)	0.000	0.89 (0.84–0.95)	0.001
Latin America	0.49 (0.45–0.52)	0.000	0.53 (0.49–0.57)	0.000
North Africa	0.63 (0.57–0.70)	0.000	0.66 (0.59–0.73)	0.000
EU and North America	0.98 (0.87–1.10)	0.719	0.99 (0.89–1.12)	0.953
Area of residence				
Urban	ref.		ref.	
Rural	0.96 (0.94–0.98)	0.002	0.93 (0.91–0.95)	0.000
Deprivation index ^3^				
Q_1_	ref.		ref.	
Q_2_	0.97 (0.94–0.99)	0.041	0.96 (0.93–0.99)	0.016
Q_3_	0.99 (0.96–1.03)	0.915	0.98 (0.95–1.01)	0.230
Q_4_	0.94 (0.91–0.97)	0.000	0.93 (0.90–0.96)	0.000

^1^ Odds ratio; ^2^ Adjusted odds ratios for sex and age; ^3^ Deprivation index of the residence area according to 26 socio-economic indicators and categorized from least (Q_1_) to most (Q_4_) deprived.

**Table 3:** Socio-demographic and clinical characteristics of patients with psoriasis in the EpiChron Cohort in 2019.

Characteristics	Men	Women	Total
N (%)	16,866 (54.10)	14,312 (45.90)	31,178 (100)
Mean age, years (SD ^1^)	47.19 (17.81)	45.77 (19.04)	46.54 (18.40)
Age group, years (n, %)			
0–11	310 (1.84)	395 (2.76)	705 (2.26)
12–17	496 (2.94)	650 (4.54)	1146 (3.68)
18–44	6747 (40.00)	5755 (40.21)	12,502 (40.10)
45–64	6281 (37.24)	4965 (34.69)	11,246 (36.07)
≥65	3032 (17.98)	2547 (17.80)	5579 (17.89)
Nationality (n, %)			
Spain	15,450 (91.60)	13,079 (91.38)	28,529 (91.50)
Eastern Europe	519 (3.08)	531 (3.71)	1050 (3.37)
Asia	81 (0.48)	42 (0.29)	123 (0.39)
North Africa	240 (1.42)	117 (0.82)	357 (1.15)
Sub-Saharan Africa	53 (0.31)	15 (0.10)	68 (0.22)
Latin America	362 (2.15)	397 (2.77)	759 (2.43)
EU and North America	161 (0.95)	131 (0.92)	292 (0.94)
Area of residence ^2^			
Urban (n, %)	10,013 (59.37)	8961 (62.61)	18,974 (60.86)
Deprivation index ^3^ (n, %)			
Q_1_	4527 (26.84)	3945 (27.56)	8472 (27.17)
Q_2_	4072 (24.14)	3504 (24.48)	7576 (24.30)
Q_3_	3643 (21.60)	3067 (21.43)	6710 (21.52)
Q_4_	4624 (27.42)	3794 (26.51)	8418 (27.00)
Number of chronic diseases (mean, s.d.)	3.47 (2.86)	4.08 (3.06)	3.75 (2.97)
Multimorbidity, yes (n, %)	11,870 (70.38)	11,128 (77.75)	22,998 (73.76)

^1^ Standard deviation; ^2^ Versus rural; ^3^ Deprivation index of the residence area according to 26 socio-economic indicators and categorized from least least (Q_1_) to most (Q_4_) deprived.

**Table 4:** Prevalence of chronic comorbidities and of specific acute conditions (in italics) in patients with psoriasis in the EpiChron Cohort in 2019 (n = 31,178). Logistic regression models were used to calculate odds ratios (OR) of prevalence for each comorbidity (dependent variable) according to the presence or absence of psoriasis (independent variable). ICD-9-CM diagnoses included in each category of CCS code can be found at: https://hcup-us.ahrq.gov/toolssoftware/ccs/AppendixASingleDX.txt (accessed on 9 December 2023) [1,3].

	Comorbidity	Prevalence	Crude OR	Adjusted OR ^1^	*p*-Value ^2^
Respiratory comorbidities					
*EAR11*	*Acute upper respiratory tract infection*	15,318 (49.13)	1.00 (0.98–1.03)	1.07 (1.05–1.10)	0.000
*EAR09*	*Other upper respiratory diseases (Pharyngitis and tonsillitis)*	4473 (14.35)	0.96 (0.93–0.99)	1.05 (1.02–1.09)	0.002
D128	Asthma	2006 (6.43)	0.86 (0.82–0.90)	0.98 (0.94–1.03)	0.456
D127	Chronic obstructive pulmonary disease and bronchiectasis	1563 (5.01)	1.39 (1.32–1.47)	1.23 (1.17–1.30)	0.000
*vrsup*	*infecc. estreptocócicas de vías resp. superiores*	1232 (3.95)	0.90 (0.85–0.95)	1.26 (1.19–1.34)	0.000
D133	Other lower respiratory disease	36 (0.12)	0.96 (0.69–1.34)	0.92 (0.66–1.28)	0.632
D56	Cystic fibrosis	14 (0.04)	1.89 (1.10–3.24)	2.15 (1.25–3.69)	0.005
D132	Lung disease due to external agents	8 (0.03)	1.46 (0.72–2.96)	1.26 (0.62–2.57)	0.514
Cardiac-Cardiovascular comorbidities					
D53	Disorders of lipid metabolism	11,182 (35.87)	1.17 (1.15–1.20)	1.08 (1.06–1.11)	0.000
G7_1	Hypertension	11,068 (35.50)	1.09 (1.07–1.12)	0.98 (0.96–1.01)	0.184
D106	Cardiac dysrhythmias	1860 (5.97)	0.94 (0.89–0.98)	0.88 (0.83–0.92)	0.000
D100	Acute myocardial infarction	1165 (3.74)	1.11 (1.04–1.17)	0.98 (0.93–1.05)	0.625
D108	Congestive heart failure; nonhypertensive	778 (2.50)	0.79 (0.73–0.85)	0.79 (0.74–0.86)	0.000
D109	Acute cerebrovascular disease	687 (2.20)	0.78 (0.73–0.85)	0.76 (0.70–0.82)	0.000
D112	Transient cerebral ischemia	512 (1.64)	0.83 (0.76–0.91)	0.83 (0.76–0.90)	0.000
D105	Conduction disorders	408 (1.31)	1.03 (0.94–1.14)	0.94 (0.85–1.04)	0.225
D114	Peripheral and visceral atherosclerosis	358 (1.15)	1.31 (1.18–1.46)	1.10 (0.99–1.22)	0.079
D117	Other circulatory disease	339 (1.09)	1.10 (0.99–1.23)	1.07 (0.96–1.19)	0.218
D96	Heart valve disorders	336 (1.08)	0.99 (0.89–1.10)	0.96 (0.86–1.07)	0.515
D97	Peri-; endo-; and myocarditis; cardiomyopathy (except that caused by tuberculosis or sexually transmitted disease)	194 (0.62)	1.07 (0.93–1.24)	0.97 (0.84–1.12)	0.727
D115	Aortic; peripheral; and visceral artery aneurysms	118 (0.38)	1.35 (1.12–1.62)	1.14 (0.95–1.37)	0.170
D104	Other and ill-defined heart disease	117 (0.38)	0.95 (0.79–1.14)	0.89 (0.75–1.07)	0.241
D121	Other diseases of veins and lymphatics	90 (0.29)	1.17 (0.95–1.45)	1.27 (1.03–1.57)	0.027
D116	Aortic and peripheral arterial embolism or thrombosis	47 (0.15)	1.07 (0.79–1.42)	0.99 (0.74–1.33)	0.971
D103	Pulmonary heart disease	43 (0.14)	1.06 (0.78–1.43)	1.01 (0.75–1.37)	0.952
D111	Other and ill-defined cerebrovascular disease	36 (0.12)	0.83 (0.60–1.16)	0.83 (0.60–1.16)	0.279
D213	Cardiac and circulatory congenital anomalies	25 (0.08)	0.44 (0.29–0.66)	0.84 (0.57–1.26)	0.406
D107	Cardiac arrest and ventricular fibrillation	5 (0.02)	1.34 (0.55–3.27)	1.18 (0.48–2.90)	0.709
D248	Gangrene	4 (0.01)	0.97 (0.36–2.62)	0.93 (0.35–2.53)	0.895
D183	Hypertension complicating pregnancy; childbirth and the puerperium	2 (0.01)	0.28 (0.07–1.14)	0.37 (0.09–1.50)	0.166
Endocrine-Nutritional comorbidities					
G3_11	Other nutritional; endocrine; and metabolic disorders	6794 (21.79)	1.12 (1.09–1.15)	1.06 (1.03–1.09)	0.000
G3_23	Diabetes Mellitus	3989 (12.79)	1.15 (1.11–1.18)	1.05 (1.02–1.09)	0.004
D300	Obesity	3807 (12.21)	1.24 (1.19–1.28)	1.23 (1.19–1.28)	0.000
D48	Thyroid disorders	3650 (11.71)	0.97 (0.94–1.01)	1.06 (1.02–1.09)	0.002
D51	Other endocrine disorders	96 (0.31)	0.86 (0.70–1.05)	0.87 (0.72–1.07)	0.205
D52	Nutritional deficiencies	25 (0.08)	0.93 (0.62–1.38)	0.95 (0.64–1.41)	0.800
Mental-Psychological comorbidities					
D651	Anxiety disorders	4984 (15.99)	1.06 (1.03–1.09)	1.12 (1.09–1.16)	0.000
D657	Depression and mood disorders	4669 (14.98)	1.06 (1.03–1.10)	1.10 (1.06–1.13)	0.000
D653	Delirium, dementia, and amnestic and other cognitive disorders	1539 (4.94)	0.75 (0.72–0.79)	0.75 (0.71–0.79)	0.000
D670	Miscellaneous mental health disorders	786 (2.52)	0.83 (0.77–0.89)	0.92 (0.86–0.99)	0.032
D660	Alcohol-related disorders	578 (1.85)	1.43 (1.31–1.55)	1.26 (1.16–1.37)	0.000
D661	Substance-related disorders	168 (0.54)	0.85 (0.73–0.99)	0.94 (0.80–1.09)	0.410
D659	Schizophrenia and other psychotic disorders	163 (0.52)	0.82 (0.70–0.96)	0.78 (0.67–0.92)	0.003
D652	Attention-deficit, conduct, and disruptive behavior disorders	115 (0.37)	0.65 (0.54–0.78)	0.63 (0.52–0.75)	0.000
D658	Personality disorders	110 (0.35)	0.93 (0.77–1.13)	0.93 (0.77–1.12)	0.447
D304	Sleeping disorders	67 (0.21)	0.58 (0.45–0.73)	0.82 (0.64–1.05)	0.112
D305	Somatization and hypochondria disorders	27 (0.09)	1.03 (0.71–1.52)	1.09 (0.74–1.60)	0.654
Reumatological-Traumatological comorbidities					
D203	Osteoarthritis	4200 (13.47)	1.06 (1.02–1.09)	1.07 (1.04–1.11)	0.000
D205	Spondylosis; intervertebral disc disorders; other back problems	2713 (8.70)	1.24 (1.19–1.29)	1.21 (1.17–1.26)	0.000
D206	Osteoporosis	2029 (6.51)	0.92 (0.88–0.97)	1.06 (1.01–1.11)	0.018
D54	Gout and other crystal arthropathies	1048 (3.36)	1.37 (1.29–1.46)	1.14 (1.06–1.21)	0.000
D208	Acquired foot deformities	812 (2.60)	1.04 (0.97–1.12)	1.12 (1.04–1.20)	0.002
D225	Joint disorders and dislocations; trauma-related	452 (1.45)	1.25 (1.14–1.38)	1.29 (1.18–1.42)	0.000
D204	Other non-traumatic joint disorders	396 (1.27)	1.24 (1.12–1.37)	1.21 (1.09–1.33)	0.000
D202	Rheumatoid arthritis and related disease	382 (1.23)	1.39 (1.25–1.54)	1.42 (1.28–1.57)	0.000
D212	Other bone disease and musculoskeletal deformities	249 (0.80)	1.31 (1.15–1.48)	1.39 (1.22–1.57)	0.000
D301	Other microcrystalline arthritis	127 (0.41)	1.31 (1.10–1.57)	1.18 (0.99–1.41)	0.066
D211	Other connective tissue disease	125 (0.40)	1.18 (0.99–1.41)	1.22 (1.02–1.46)	0.029
D210	Systemic lupus erythematosus and connective tissue disorders	124 (0.40)	1.12 (0.93–1.34)	1.22 (1.02–1.46)	0.029
D201	Infective arthritis and osteomyelitis (except that caused by tuberculosis or sexually transmitted disease)	34 (0.11)	0.89 (0.63–1.25)	0.84 (0.60–1.18)	0.319
Neurological comorbidities					
D84	Headache; including migraine	2602 (8.35)	0.83 (0.79–0.85)	0.95 (0.91–0.98)	0.011
D95	Other nervous system disorders	1355 (4.35)	1.23 (1.16–1.30)	1.28 (1.21–1.35)	0.000
D83	Epilepsy; convulsions	320 (1.03)	0.87 (0.78–0.98)	0.88 (0.78–0.98)	0.021
D81	Other hereditary and degenerative nervous system conditions	221 (0.71)	1.24 (1.08–1.42)	1.27 (1.11–1.45)	0.001
D79	Parkinson’s disease	200 (0.64)	0.74 (0.64–0.85)	0.72 (0.63–0.83)	0.000
D82	Paralysis	108 (0.35)	0.84 (0.69–1.02)	0.80 (0.66–0.97)	0.023
D80	Multiple sclerosis	48 (0.15)	1.03 (0.77–1.37)	1.14 (0.85–1.51)	0.382
D216	Nervous system congenital anomalies	27 (0.09)	0.67 (0.46–0.98)	0.69 (0.47–1.02)	0.063
D78	Other CNS infection and poliomyelitis	4 (0.01)	0.75 (0.28–2.01)	0.77 (0.29–2.08)	0.611
D113	Late effects of cerebrovascular disease	3 (0.01)	1.58 (0.49–5.03)	1.56 (0.49–4.96)	0.453
D227	Spinal cord injury	2 (0.01)	0.60 (0.15–2.43)	0.54 (0.13–2.18)	0.387
Otorhinolaryngological comorbidities					
*EAR07*	*Wax in ear*	2716 (8.71)	1.16 (1.11–1.21)	1.11 (1.06–1.15)	0.000
D94	Other ear and sense organ disorders	2281 (7.32)	1.07 (1.02–1.12)	1.01 (0.97–1.06)	0.573
*EAR01*	*Otitis media*	1716 (5.50)	0.91 (0.86–0.95)	1.13 (1.08–1.19)	0.000
*EAR06*	*Otitis externa*	219 (0.70)	1.62 (1.42–1.86)	1.65 (1.44–1.88)	0.000
D93	Conditions associated with dizziness or vertigo	60 (0.19)	1.18 (0.91–1.53)	1.19 (0.92–1.54)	0.184
Ocular comorbidities					
D86	Cataract	2437 (7.82)	1.04 (0.99–1.08)	1.02 (0.97–1.06)	0.445
D89	Blindness and vision defects	2137 (6.85)	0.84 (0.81–0.88)	0.89 (0.85–0.93)	0.000
D88	Glaucoma	1685 (5.40)	1.06 (1.01–1.11)	1.02 (0.97–1.07)	0.422
D87	Retinal detachments; defects; vascular occlusion; and retinopathy	455 (1.46)	1.03 (0.94–1.13)	0.99 (0.91–1.09)	0.935
D91	Other eye disorders	455 (1.46)	1.13 (1.03–1.25)	1.14 (1.04–1.25)	0.006
*EYE07*	*Conjunctivitis, keratitis*	411 (1.32)	1.22 (1.10–1.34)	1.18 (1.07–1.30)	0.001
D90	Inflammation; infection of eye (except that caused by tuberculosis or sexually transmitteddisease)	14 (0.04)	1.07 (0.63–1.83)	1.15 (0.67–1.96)	0.607
Urological-nephrological comorbidities					
D163	Genitourinary symptoms and ill-defined conditions	2221 (7.12)	0.76 (0.73–0.79)	0.78 (0.75–0.82)	0.000
D164	Hyperplasia of prostate	1604 (5.14)	1.23 (1.17–1.30)	0.96 (0.91–1.02)	0.212
D158	Chronic kidney disease	1231 (3.95)	0.91 (0.86–0.97)	0.89 (0.84–0.95)	0.000
D166	Other male genital disorders	318 (1.02)	0.93 (0.84–1.05)	0.94 (0.84–1.05)	0.269
D165	Inflammatory conditions of male genital organs	237 (0.76)	0.99 (0.87–1.13)	1.01 (0.89–1.16)	0.816
D159	Urinary tract infections	67 (0.21)	0.91 (0.71–1.16)	0.99 (0.78–1.23)	0.972
D215	Genitourinary congenital anomalies	62 (0.20)	0.47 (0.36–0.60)	0.62 (0.48–0.79)	0.000
D162	Other diseases of bladder and urethra	25 (0.08)	0.94 (0.63–1.40)	0.90 (0.61–1.34)	0.619
D156	Nephritis; nephrosis; renal sclerosis	22 (0.07)	1.01 (0.66–1.55)	0.98 (0.64–1.50)	0.925
Gynaecological comorbidities					
D171	Menstrual disorders	2174 (6.97)	0.86 (0.82–0.89)	1.16 (1.11–1.22)	0.000
D173	Menopausal disorders	1294 (4.15)	1.09 (1.03–1.16)	1.28 (1.22–1.36)	0.000
D170	Prolapse of female genital organs	194 (0.62)	0.88 (0.76–1.01)	1.01 (0.88–1.17)	0.830
D174	Female infertility	137 (0.44)	0.70 (0.59–0.84)	0.91 (0.76–1.08)	0.271
D169	Endometriosis	116 (0.37)	0.98 (0.81–1.18)	1.19 (0.99–1.44)	0.058
D175	Other female genital disorders	101 (0.32)	0.71 (0.58–0.87)	0.90 (0.74–1.10)	0.327
Digestive-Hepatological comorbidities					
D138	Esophageal disorders	1161 (3.72)	1.17 (1.11–1.25)	1.15 (1.08–1.22)	0.000
D151	Other liver diseases	849 (2.72)	1.57 (1.46–1.68)	1.45 (1.35–1.55)	0.000
D146	Diverticulosis and diverticulitis	715 (2.29)	1.21 (1.13–1.31)	1.19 (1.10–1.28)	0.000
D155	Other gastrointestinal disorders	622 (2.00)	1.00 (0.93–1.09)	1.03 (0.95–1.12)	0.430
D6	Hepatitis	277 (0.89)	1.16 (1.03–1.30)	1.13 (0.99–1.27)	0.052
D144	Regional enteritis and ulcerative colitis	243 (0.78)	1.51 (1.33–1.72)	1.50 (1.32–1.71)	0.000
D152	Pancreatic disorders (not diabetes)	104 (0.33)	1.04 (0.85–1.26)	0.95 (0.78–1.16)	0.612
D302	Eating disorders	81 (0.26)	0.90 (0.72–1.13)	1.17 (0.94–1.46)	0.167
D149	Biliary tract disease	27 (0.09)	1.11 (0.76–1.63)	1.05 (0.72–1.55)	0.788
D214	Digestive congenital anomalies	21 (0.07)	0.29 (0.19–0.45)	0.85 (0.55–1.30)	0.452
Haematological-Immunological comorbidities					
D62	Coagulation and hemorrhagic disorders	1132 (3.63)	0.90 (0.85–0.96)	0.85 (0.80–0.90)	0.000
D63	Diseases of white blood cells	373 (1.20)	0.95 (0.86–1.06)	0.98 (0.88–1.08)	0.669
D59	Deficiency and other anemia	74 (0.24)	0.81 (0.65–1.02)	0.90 (0.72–1.14)	0.382
D57	Immunity disorders	28 (0.09)	1.12 (0.77–1.63)	1.08 (0.74–1.58)	0.674
D61	Sickle cell anemia	3 (0.01)	0.36 (0.11–1.12)	0.40 (0.13–1.24)	0.112
Dermatological comorbidities					
*SKN04*	*Acne*	583 (1.87)	0.66 (0.61–0.72)	1.02 (0.94–1.11)	0.657
D199	Chronic ulcer of skin	542 (1.74)	0.70 (0.64–0.76)	0.72 (0.65–0.78)	0.000
*piel*	*infecc. local no especificada de la piel y tejido subcutáneo*	125 (0.40)	1.35 (1.13–1.61)	1.39 (1.16–1.66)	0.000
Non-specific organ infective comorbidities					
D5	HIV infection	73 (0.23)	0.79 (0.63–1.00)	0.80 (0.64–1.01)	0.067
D7	Viral infection	38 (0.12)	0.98 (0.71–1.35)	1.16 (0.84–1.61)	0.358
D8	Other infections; including parasitic	16 (0.05)	1.44 (0.87–2.37)	1.44 (0.87–2.37)	0.155
D1	Tuberculosis	10 (0.03)	2.48 (1.30–4.71)	2.36 (1.24–4.49)	0.009
Miscellaneous comorbidities					
G2	Neoplasms	1596 (5.12)	0.97 (0.92–1.02)	0.90 (0.86–0.95)	0.000
D259	Residual codes; unclassified	1016 (3.26)	1.62 (1.52–1.72)	1.44 (1.35–1.53)	0.000
D303	Sexual disorders	790 (2.53)	1.40 (1.30–1.50)	1.19 (1.11–1.28)	0.000
D253	Allergic reactions	747 (2.40)	0.45 (0.42–0.49)	0.82 (0.76–0.88)	0.000
D209	Other acquired deformities	571 (1.83)	0.76 (0.69–0.82)	0.96 (0.88–1.05)	0.415
D167	Nonmalignant breast conditions	418 (1.34)	0.96 (0.87–1.06)	1.17 (1.06–1.29)	0.001
D217	Other congenital anomalies	261 (0.84)	0.56 (0.50–0.64)	0.91 (0.81–1.03)	0.155
D655	Disorders usually diagnosed in infancy, childhood, or adolescence	100 (0.32)	0.28 (0.23–0.35)	0.44 (0.36–0.54)	0.000
D654	Developmental disorders	34 (0.11)	0.55 (0.39–0.77)	0.64 (0.45–0.89)	0.009
D252	Malaise and fatigue	20 (0.06)	1.36 (0.87–2.13)	1.49 (0.95–2.33)	0.079

^1^ odds ratios adjusted by sex and age; ^2^ *p*-values for the adjusted OR.

## Data Availability

The data used in this study cannot be publicly shared, because of restrictions imposed by the Aragon Health Sciences Institute (IACS) and asserted by the Clinical Research Ethics Committee of Aragon (CEICA, ceica@aragon.es). The authors can establish future collaborations with other groups based on the same data. Potential collaborations should be addressed to the Principal Investigator of the EpiChron Group, Alexandra Prados-Torres, sprados.iacs@aragon.es.

## References

[1] Ferrándiz C., Carrascosa J.M., Toro M. (2014). Prevalencia de la psoriasis en España en la era de los agentes biológicos. Actas Dermosifiliogr..

[2] Herraiz M.R., Rodríguez J.J.P. (2016). La genética de la psoriasis. Med. Cutan. Ibero. Lat. Am..

[3] Lebwohl M. (2003). Psoriasis. Lancet.

[4] Parisi R., Symmons D.P.M., Griffiths C.E.M., Ashcroft D.M. (2013). Global epidemiology of psoriasis: A systematic review of incidence and prevalence. J. Investig. Dermatol..

[5] Gudjonsson J.E., Elder J.T. (2007). Psoriasis: Epidemiology. Clin. Dermatol..

[6] Dermatology E-Book—Jean L. Bolognia, Julie V. Schaffer, Lorenzo Cerroni—Google Libros. https://books.google.es/books?id=Ufw6DwAAQBAJ&printsec=frontcover&dq=bolognia+4+edition&hl=es&sa=X&ved=2ahUKEwjA96T177rwAhUKoRQKHb7IDs4Q6AEwAnoECAYQAg#v=onepage&q=bolognia%204%20edition&f=false.

[7] Yamazaki F. (2021). Psoriasis: Comorbidities. J. Dermatol..

[8] Alajmi R.S., Alamoudi S.M., Alabbasi A.A., Alwagdani A., Alraddadi A.A., Alamri A. (2021). Patterns of Comorbidities in Psoriasis Patients: A Cross-Sectional Study. Cureus.

[9] Dauden E., Blasco A.J., Bonanad C., Botella R., Carrascosa J.M., González-Parra E., Jodar E., Joven B., Lázaro P., Olveira A. (2018). Position statement for the management of comorbidities in psoriasis. J. Eur. Acad. Dermatol. Venereol..

[10] Badaoui A., Tounian P., Mahé E. (2019). Psoriasis and metabolic and cardiovascular comorbidities in children: A systematic review. Arch. De Pediatr..

[11] Horreau C., Pouplard C., Brenaut E., Barnetche T., Misery L., Cribier B., Jullien D., Aractingi S., Aubin F., Joly P. (2013). Cardiovascular morbidity and mortality in psoriasis and psoriatic arthritis: A systematic literature review. J. Eur. Acad. Dermatol. Venereol..

[12] Gilaberte Y., Pérez-Gilaberte J.B., Poblador-Plou B., Bliek-Bueno K., Gimeno-Miguel A., Prados-Torres A. (2020). Prevalence and Comorbidity of Atopic Dermatitis in Children: A Large-Scale Population Study Based on Real-World Data. J. Clin. Med..

[13] Elixhauser A., Steiner C., Palmer L. Clinical Classifications Software (CCS), 2009. Agency for Healthcare Research and Quality. http://www.hcup-us.ahrq.gov/toolssoftware/ccs/ccs.jsp.

[14] Chronic Condition Indicator (CCI) for ICD-9-CM. https://www.hcup-us.ahrq.gov/toolssoftware/chronic/chronic.jsp.

[15] Chang Y.T., Chen T.J., Liu P.C., Chen Y.C., Chen Y.J., Huang Y.L., Jih J.S., Chen C.C., Lee D.D., Wang W.J. (2009). Epidemiological study of psoriasis in the national health insurance database in Taiwan. Acta Derm. Venereol..

[16] Falk E.S., Vandbakk O. (1993). Prevalence of psoriasis in a Norwegian Lapp population. Acta Derm. Venereol. Suppl..

[17] Barisić-Drusko V., Paljan D., Kansky A., Vujasinović S. (1989). Prevalence of psoriasis in Croatia. Acta Derm. Venereol. Suppl..

[18] Gelfand J.M., Stern R.S., Nijsten T., Feldman S.R., Thomas J., Kist J., Rolstad T., Margolis D.J. (2005). The prevalence of psoriasis in African Americans: Results from a population-based study. J. Am. Acad. Dermatol..

[19] Schlander M., Schwarz O., Viapiano M., Bonauer N. (2008). PSS21 Administrative Prevalence of Psoriasis in Germany. Value Health.

[20] Plunkett A., Marks R. (1998). A review of the epidemiology of psoriasis vulgaris in the community. Australas. J. Dermatol..

[21] Brandrup F., Green A. (1981). The prevalence of psoriasis in Denmark. Acta Derm. Venereol..

[22] Jesić S., Hosovski E. (1994). Prevalence of psoriasis vulgaris in West Serbia. Srp. Arh. Celok. Lek..

[23] Braathen L.R., Botten G., Bjerkedal T. (1989). Prevalence of psoriasis in Norway. Acta Derm. Venereol. Suppl..

[24] Miao C., Li J., Zhang X. (2019). Obesity and dyslipidemia in patients with psoriasis: A case-control study. Medicine.

[25] Salihbegovic E.M., Hadzigrahic N., Suljagic E., Kurtalic N., Hadzic J., Zejcirovic A. (2015). Psoriasis and Dyslipidemia. Mater. Socio-Medica.

[26] Jensen P., Skov L. (2017). Psoriasis and Obesity. Dermatology.

[27] Pérez-rodrigo C., Bartrina J.A., Majem L.S., Moreno B., Rubio A.D. (2006). Epidemiology of Obesity in Spain. Diet. Guidel. Strateg. Prev..

[28] Wu C.Y., Hu H.Y., Li C.P., Chou Y.J., Chang Y.T. (2018). Comorbidity profiles of psoriasis in Taiwan: A latent class analysis. PLoS ONE.

[29] Cho S.I., Kim Y.E., Jo S.J. (2021). Association of metabolic comorbidities with pediatric psoriasis: A systematic review and meta-analysis. Ann. Dermatol..

[30] Mamizadeh M., Tardeh Z., Azami M. (2019). The association between psoriasis and diabetes mellitus: A systematic review and meta-analysis. Diabetes Metab. Syndr..

[31] Armstrong A.W., Harskamp C.T., Armstrong E.J. (2013). Psoriasis and the risk of diabetes mellitus: A systematic review and meta-analysis. JAMA Dermatol..

[32] Bellinato F., Gisondi P., Mantovani A., Girolomoni G., Targher G. (2022). Risk of non-alcoholic fatty liver disease in patients with chronic plaque psoriasis: An updated systematic review and meta-analysis of observational studies. J. Endocrinol. Investig..

[33] Alinaghi F., Calov M., Kristensen L.E., Gladman D.D., Coates L.C., Jullien D., Gottlieb A.B., Gisondi P., Wu J.J., Thyssen J.P. (2019). Prevalence of psoriatic arthritis in patients with psoriasis: A systematic review and meta-analysis of observational and clinical studies. J. Am. Acad. Dermatol..

[34] Lubrano E., Scriffignano S., Perrotta F.M. (2020). Multimorbidity and comorbidity in psoriatic arthritis–a perspective. Expert. Rev. Clin. Immunol..

[35] Kamalaraj N., El-Haddad C., Hay P., Pile K. (2019). Systematic review of depression and anxiety in psoriatic arthritis. Int. J. Rheum. Dis..

[36] Brenaut E., Horreau C., Pouplard C., Barnetche T., Paul C., Richard M.A., Joly P., Le Maître M., Aractingi S., Aubin F. (2013). Alcohol consumption and psoriasis: A systematic literature review. J. Eur. Acad. Dermatol. Venereol..

[37] Zhao S.S., Miller N., Harrison N., Duffield S.J., Dey M., Goodson N.J. (2020). Systematic review of mental health comorbidities in psoriatic arthritis. Clin. Rheumatol..

[38] Molina-Leyva A., Salvador-Rodriguez L., Martinez-Lopez A., Ruiz-Carrascosa J.C., Arias-Santiago S. (2019). Association between Psoriasis and Sexual and Erectile Dysfunction in Epidemiologic Studies: A Systematic Review. JAMA Dermatol..

[39] Sources D., Selection S., Extraction D., Outcomes M. (2020). Prevalence, Incidence, and Risk of Cancer in Patients With Psoriasis and Psoriatic Arthritis A Systematic Review and Meta-analysis. JAMA Dermatol..

[40] Ng C.Y., Huang Y.H., Chu C.F., Wu T.C., Liu S.H. (2017). Risks for Staphylococcus aureus colonization in patients with psoriasis: A systematic review and meta-analysis. Br. J. Dermatol..

[41] Garcia-Doval I., Cohen A.D., Cazzaniga S., Feldhamer I., Addis A., Carretero G., Ferrándiz C., Stern R.S., Naldi L., Network P. (2017). Risk of serious infections, cutaneous bacterial infections, and granulomatous infections in patients with psoriasis treated with anti-tumor necrosis factor agents versus classic therapies: Prospective meta-analysis of Psonet registries. J. Am. Acad. Dermatol..

[42] Sánchez-Moya A.I., García-Doval I., Carretero G., Sánchez-Carazo J., Ferrandiz C., Herrera Ceballos E., Alsina M., Ferrán M., López-Estebaranz J.L., Gómez-García F. (2013). Latent tuberculosis infection and active tuberculosis in patients with psoriasis: A study on the incidence of tuberculosis and the prevalence of latent tuberculosis disease in patients with moderate–severe psoriasis in Spain. BIOBADADERM registry. J. Eur. Acad. Dermatol. Venereol..

[43] Chowdhary R., Tan S.L., Pavesi G., Jin J., Dong D., Mathur S.K., Burkart A., Narang V., Glurich I., Raby B.A. (2012). A Database of Annotated Promoters of Genes Associated with Common Respiratory and Related Diseases. Am. J. Respir. Cell Mol. Biol..

[44] Chen H., Zhang J., He Y., Lv Z., Liang Z., Chen J., Li P., Liu J., Yang H., Tao A. (2022). Exploring the Role of Staphylococcus aureus in Inflammatory Diseases. Toxins.

[45] Goerke C., Wolz C. (2010). Adaptation of Staphylococcus aureus to the cystic fibrosis lung. Int. J. Med. Microbiol..

[46] Blauvelt A., Chiricozzi A. (2018). The Immunologic Role of IL-17 in Psoriasis and Psoriatic Arthritis Pathogenesis. Clin. Rev. Allergy Immunol..

[47] Benjamin C.M., Clague R.B. (1990). Psoriatic or cystic fibrosis arthropathy? Difficulty with diagnosis and management. Rheumatology.

[48] Schröder J.M., Harder J. (1999). Human beta-defensin-2. Int. J. Biochem. Cell Biol..

[49] Underwood M.A., Bevins C.L. (2010). Defensin-Barbed Innate Immunity: Clinical Associations in the Pediatric Population. Pediatrics.

[50] van de Kerkhof P.C.M., Murphy G.M., Austad J., Ljungberg A., Cambazard F., Duvold L.B. (2007). Psoriasis of the face and flexures. J. Dermatolog. Treat..

[51] Antonelli P.J., Schultz G.S., Cantwell J.S., Sundin D.J., Pemberton P.A., Barr P.J. (2005). Inflammatory proteases in chronic otitis externa. Laryngoscope.

[52] Lee H.Y., Andalibi A., Webster P., Moon S.K., Teufert K., Kang S.H., Li J.D., Nagura M., Ganz T., Lim D.J. (2004). Antimicrobial activity of innate immune molecules against Streptococcus pneumoniae, Moraxella catarrhalis and nontypeable Haemophilus influenzae. BMC Infect. Dis..

[53] Jin Shin D., Gan-Undram S., Jin Kim S., Joon Jun Y., Jung Im G., Hyun Jung H. (2006). Expression of β-defensins in the tubotympanum of experimental otitis media. Acta Otolaryngol..

